# A time-scale representation of EEG Higuchi fractal dimension

**DOI:** 10.1038/s41598-026-53819-3

**Published:** 2026-05-29

**Authors:** Safoora Masoumirad, Laura Päeske, Jaanus Lass, Maie Bachmann

**Affiliations:** Biosignal Processing Laboratory, Department of Health Technologies, School of Information Technologies, Tallinn University of Technology, Ehitajate tee 5, Tallinn, 19086 Estonia

**Keywords:** Electroencephalography, Nonlinear analysis, Higuchi’s fractal dimension, Time-scale representation, Sampling frequency, Parameter *k*_*max*_, Biological techniques, Computational biology and bioinformatics, Engineering, Neuroscience

## Abstract

Higuchi Fractal Dimension (HFD) is a widely used nonlinear metric with extensive application in biological signal analysis, including electroencephalography (EEG) signal processing. HFD relies on a single free parameter, *k*_*max*_, which is the maximum scale in samples, used to assess the signal’s self-similarity. While fractal dimension estimation is independent of sampling frequency (*Fs*) in ideally fractal signals, EEG signals do not exhibit ideal fractality, also making the HFD estimates sensitive to both parameter *k*_*max*_ and sampling frequency. As a result, HFD results reported across studies are difficult to compare with different parameter settings. In this work, we emphasize the need to account for the sampling frequency and present the HFD results in terms of the maximum time interval, *t*_*max*_ *= k*_*max*_*/Fs*, rather than *k*_*max*_, thereby facilitating comparisons across studies. Additionally, we demonstrate that the choice of maximum time interval (*t*_*max*_) determines the frequency range that HFD focuses on, improving the interpretation of the results and enabling a more informed parameter selection.

## Introduction

Brain functions as a complex, nonlinear system^1^, making nonlinear analysis particularly suited for revealing the underlying dynamics of electroencephalographic (EEG) signals that may not be fully captured by traditional linear methods. Higuchi’s algorithm^2^ is a widely used nonlinear approach that estimates the fractal dimension as a measure of complexity and self-similarity in the time domain, without the need to embed the signal in a phase space. The resulting measure, known as Higuchi’s fractal dimension (HFD), is an effective biomarker for capturing dynamic changes in a signal, revealing its underlying scaling relationships.

HFD has been applied to EEG signals for nearly three decades, with an early contribution from Inouye et al. in 1994^3^, which analyzed wakefulness and sleep cycles to provide insights into transitions between different sleep stages. Accardo et al.^4^ later demonstrated its effectiveness in short EEG segments, leading to its gradual yet broader adoption in EEG analysis, including but not limited to the study of neurodegenerative diseases, such as Alzheimer’s and Parkinson’s^5,6^, detecting seizure onsets and abnormal brain activity in epilepsy research^7,8^, distinguishing various mental disorders from healthy individuals, including depression and schizophrenia^9–11^, stress classification^12^ and monitoring the depth of anesthesia (DOA)^13^.

Despite its widespread use and promising utility, reported HFD values vary considerably: even in healthy subjects, mean HFD values range from as low as 1.02^14^ and 1.07^15^ to nearly 1.94^5^. Lower HFD values indicate a less complex signal, while values near two refer to a more complex signal or near-to-random white noise. These divergent values are influenced to some extent by individual differences in fractal dynamics^16^, by different preprocessing methods^17^, by different noise levels^4^ or hardware variability. While it has been shown that HFD is relatively independent of signal length (*N*)^4,18,19^, the choice of sampling frequency (*Fs*) and parameter *k*_*max*_ appears to have a substantial influence on the resulting HFD estimates.

Parameter *k*_*max*_ in Higuchi’s method is the only free parameter, which determines the maximum scale in samples used to estimate the fractal dimension. The HFD method looks for the fractal characteristics (scale relationships) in the signal starting from the minimum scale (consecutive samples, k = 1), incrementing up to the maximum scale set by *k*_*max*_ (see Methods). In principle, ideally fractal signals preserve scale-invariant relationships across all scales and therefore yield HFD values independent of the sampling frequency (*Fs*)^20^. However, EEG, as a physiological signal, does not exhibit ideal fractal behavior across all scales^21^. Consequently, the effects of sampling frequency and the parameter *k*_*max*_ become important in HFD analysis, yet to the best of our knowledge, they have not been investigated previously.

As an EEG signal representation is dependent on the sampling interval *dt = 1/Fs*, the maximum scale (*k*_*max*_) in Higuchi’s algorithm, expressed in samples, is directly defined in time units as *k*_*max*_*/Fs*. This is a direct consequence of discrete-time signal representation and is essential for comparing different studies using different sampling rates. Although the original publication by Higuchi^2^, analyzing the magnetic field data, indicated the maximum scale studied (*k*_*max*_), also as a maximum time interval, in seconds, as far as we know, all subsequent EEG studies focus on the free parameter, *k*_*max*_, in samples, without indicating the maximum time interval studied, and therefore, comparison across studies is not directly feasible. Consequently, the effects of *Fs* and *k*_*max*_ must be considered to ensure comparable HFD-based analyses.

Understanding the effects of these parameters requires presenting scales in consistent time units. According to the HFD method, the minimum time interval is determined by the sampling interval *dt = 1/Fs*, while the maximum time interval depends on the research group´s choice of *k*_*max*_ and sampling frequency given by *t*_*max*_ *= k*_*max*_*/Fs*. Here, *t*_*max*_ determines the largest scale evaluated by the HFD algorithm and is distinct from the signal duration (epoch length). When previous studies are re-expressed in these terms, it becomes clear that the temporal ranges sampled by the HFD algorithm vary substantially across the studies. For example, even among studies using the same EEG database with an identical sampling frequency (173.61 Hz), the minimum time interval remains fixed (≈ 0.006 s), whereas differing *k*_*max*_ values—5^22,23^, 45^24^, and 128^25^—result in different maximum time intervals of 0.029, 0.26, and 0.737 s. These differences in examined temporal ranges directly affect the resulting HFD estimates, and therefore, studies are not comparable.

**Fig. 1:**
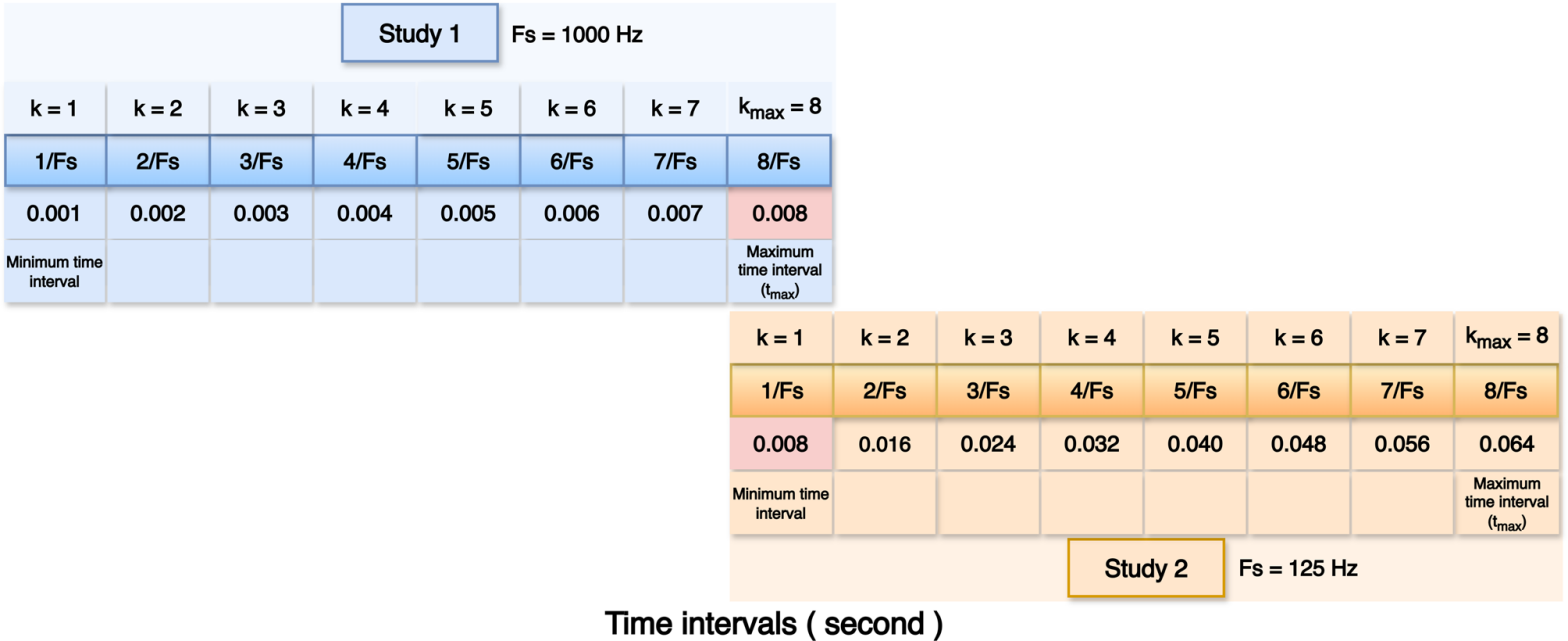
Time intervals in seconds analyzed in Studies 1 and 2. (1) Čukić et al.^14^. (2) Croce et al.^26^.

Additionally, using the same *k*_*max*_ but varying the sampling frequency results in different minimum and maximum time intervals, thereby again restricting comparability. For example, in Study 1^14^, with parameter *k*_*max*_ = 8 and *Fs* = 1000 Hz, the minimum interval is 0.001 s and the maximum interval is 0.008 s, whereas in Study 2^26^, with the same *k*_*max*_, but lower *Fs* (*Fs* = 125 Hz), the intervals become 0.008 s and 0.064 s. Thus, despite using the same *k*_*max*_, the analyzed time intervals change from [0.001, 0.008] s to [0.008, 0.064] s (see Fig. 1). Notably, the maximum interval in Study 1 equals the minimum interval in Study 2 (0.008 s), indicating that the two studies probe essentially different time-scale ranges, which is also reflected in the HFD estimates. Although both studies used the same *k*_*max*_ value, the variation in sampling frequency likely contributed to the divergence in reported HFD range for the healthy group (1.0194–1.0198 in Study 1 vs. 1.44–1.69 in Study 2).

These observations underscore the need to isolate the actual effects of sampling frequency and *k*_*max*_ from extraneous factors, including individual, noise-related, or hardware-driven variations. To achieve this, we first examine the effect of Fs and *k*_*max*_ on a representative EEG segment, illustrating how these parameters lead to differences in HFD values and limit comparability across studies. We then propose a time-scale representation of HFD to address this limitation and extend the analysis across multiple EEG segments from different subjects to indicate its consistency.

In this study, we make two main contributions. First, we propose a time-scale representation of HFD by expressing estimates in terms of the maximum time interval (*t*_*max*_ *= k*_*max*_*/Fs*), providing a foundation for comparing results across studies and encouraging more explicit reporting of the temporal scales underlying HFD analysis. Second, we demonstrate that the maximum time interval (*t*_*max*_) determines the frequency range that HFD focuses on, providing a framework for interpreting HFD results and guiding parameter selection.

## Methods

### Data

To present the effect of sampling frequency on HFD results at different *k*_*max*_ values, 240 EEG signal segments were selected from 30 healthy participants. Each segment was 10 s in duration, including both eyes-closed and eyes-open resting-state conditions.

The utilized EEG signals were recorded using the Neuroscan Synamps2 acquisition system (Compumedics, NC, USA). The signals were recorded using the International 10/20 system. Channel PZ, referenced to the average of the mastoids (M1, M2), was selected.

The raw EEG signals had a frequency band of 0.3–400 Hz at a sampling frequency of 1000 Hz. The impedance of the electrodes was below 10 kΩ. The signals were pre-processed by a digital Butterworth zero-phase lowpass filter with a cutoff frequency of 47 Hz.

The EEG signals used in this study are a subset of a dataset previously collected with the approval of the Tallinn Medical Research Ethics Committee (Approval No. 532) and published in a 2023 study^27^. The EEG signals were collected at the EEG laboratory of Tallinn University of Technology. A total of 30 healthy subjects were included in this study. Their age varied from 21 to 75 years (mean 39 ± 14 years). All methods were carried out in accordance with relevant guidelines and regulations, including the Declaration of Helsinki. The participants signed written informed consent before the study.

### Down-sampling

To study the HFD results at two different sampling frequencies, we down-sampled the signals to 250 Hz and 500 Hz, resulting in signal lengths of *N* = 2500 and *N* = 5000, respectively.

### Higuchi’s fractal dimension

Higuchi’s fractal dimension estimates the fractal dimension directly in the time domain. HFD measures how signal amplitude differences change as samples are taken at progressively larger scales, up to the maximum scale defined by *k*_*max*_. For time series representing a signal, HFD ranges from 1 for deterministic constant functions to 2 for white noise^26^. Generally, higher self-similarity and complexity result in higher HFD.

The calculation of HFD is based on the original algorithm proposed by Higuchi^2^. Original HFD^2^ starts by evaluating the scaling relationship with the minimum possible scale (the two consecutive signal samples), incrementing to the maximum scale defined by *k*_*max*_. The method seeks to identify fractal behavior over this range of scales. This process involves constructing new sequences from the time series, where each sequence is based on progressively larger scales.

Let *X(1)*,* X(2)*,* …*,* X(N)* be the N-point time series under investigation, and construct *k* new sequences as follows:

1 $$\:{X}_{k}^{m}:X\left(m\right),X\left(m+k\right),X\left(m+2k\right),\dots\:,X\left(m+\left(\frac{N-m}{k}\right).k\right)\:,\:\:\:\:\:\:m=1,\:2,\dots\:,k$$

where *m* represents the initial time point and *k*, ranging from 1 to *k*_*max*_, indicates the scales.

The length of each sequence is defined as:

2 $$\:{L}_{m}\left(\mathrm{k}\right)=\frac{1}{k}\left[\left({\sum\:}_{i=1}^{\left(\frac{N-m}{k}\right)}\left|X\left(m+ik\right)-X\left(m+(i-1\right)k\right|\right)\frac{N-1}{\left(\frac{N-m}{k}\right).k}\right]$$

where $\:\frac{(N-1)}{\left(\frac{N-m}{k}\right)k}\:$is a normalization factor. Then, the mean curve length, (*L(k))* is defined as:

3 $$\:L\left(k\right)=\frac{1}{k}\:\sum\:_{m=1}^{k}{L}_{m}\left(k\right).$$

If the mean curve length $\:\left(L\left(k\right)\right)$ behaves as a power law with respect to scale (*k*):

4 $$\:L\left(k\right)\:\propto\:\:{k}^{-FD}$$

Then, the curve under consideration is fractal with FD as the fractal dimension, which can be calculated as the slope of the least-squares linear fit to the plot of *ln (L(k))* versus *ln(1/k)*^2^.

**Fig. 2:**
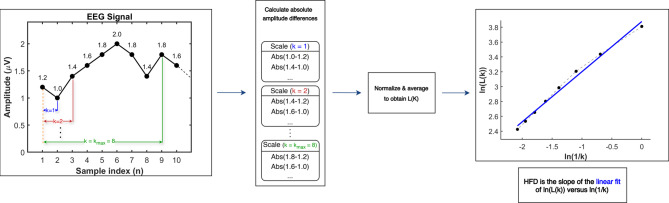
Illustration of the mechanism of Higuchi’s algorithm for an example *k*_*max*_ value of 8.

Figure 2 illustrates the mechanism of Higuchi’s algorithm for an example *k*_*max*_ value of 8. The algorithm begins at the smallest scale *(k = 1)*, where consecutive samples are compared, and their absolute amplitude differences are computed (shown in blue). At the next scale *(k = 2)*, the algorithm computes absolute differences between time points that are two samples apart (shown in red). This procedure continues with progressively larger *k* values up to the maximum scale *(k =k*_*max*_ *= 8)*, where absolute differences are calculated between time points that are eight samples apart (shown in green).

The resulting absolute differences are then normalized and averaged to obtain the mean curve length *(L(k))*. The normalization accounts for the differing number of evaluated sample pairs across scales. Finally, the slope of the least-squares linear fit to the plot of (ln(L(k), ln(1/k)) is the estimated Higuchi fractal dimension.

### Statistical analysis

Agreement between HFD estimates down-sampled to 250 Hz and 500 Hz was evaluated at matched *t*_*max*_ values across all 240 signal segments using Spearman’s rank correlation coefficient. Error magnitude was quantified using Root Mean Square Error (RMSE).

## Results

### HFD analysis using *k*_*max*_ and maximum time interval

**Fig. 3:**
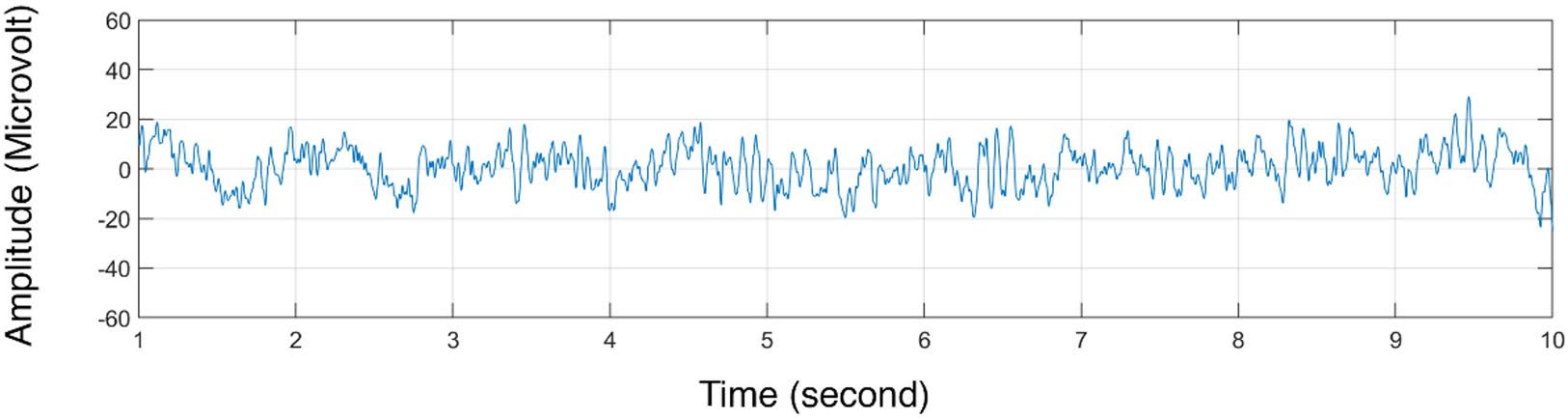
An example 10-second EEG signal selected for the analysis.

Figure 3 presents an example 10-second EEG signal selected from the dataset used for the HFD analysis.

To demonstrate the effect of sampling frequency on HFD values, we evaluated the HFD values at different *k*_*max*_ values for the example EEG signal down-sampled to 500 Hz and 250 Hz. The lower limit for *k*_*max*_ was 2. The upper limit for *k*_*max*_ was chosen to include a maximum time interval of 0.5 s. Therefore, the maximum value of *k*_*max*_ for *Fs* = 250 Hz is 125 samples, and for *Fs* = 500 Hz is 250 samples.

**Fig. 4:**
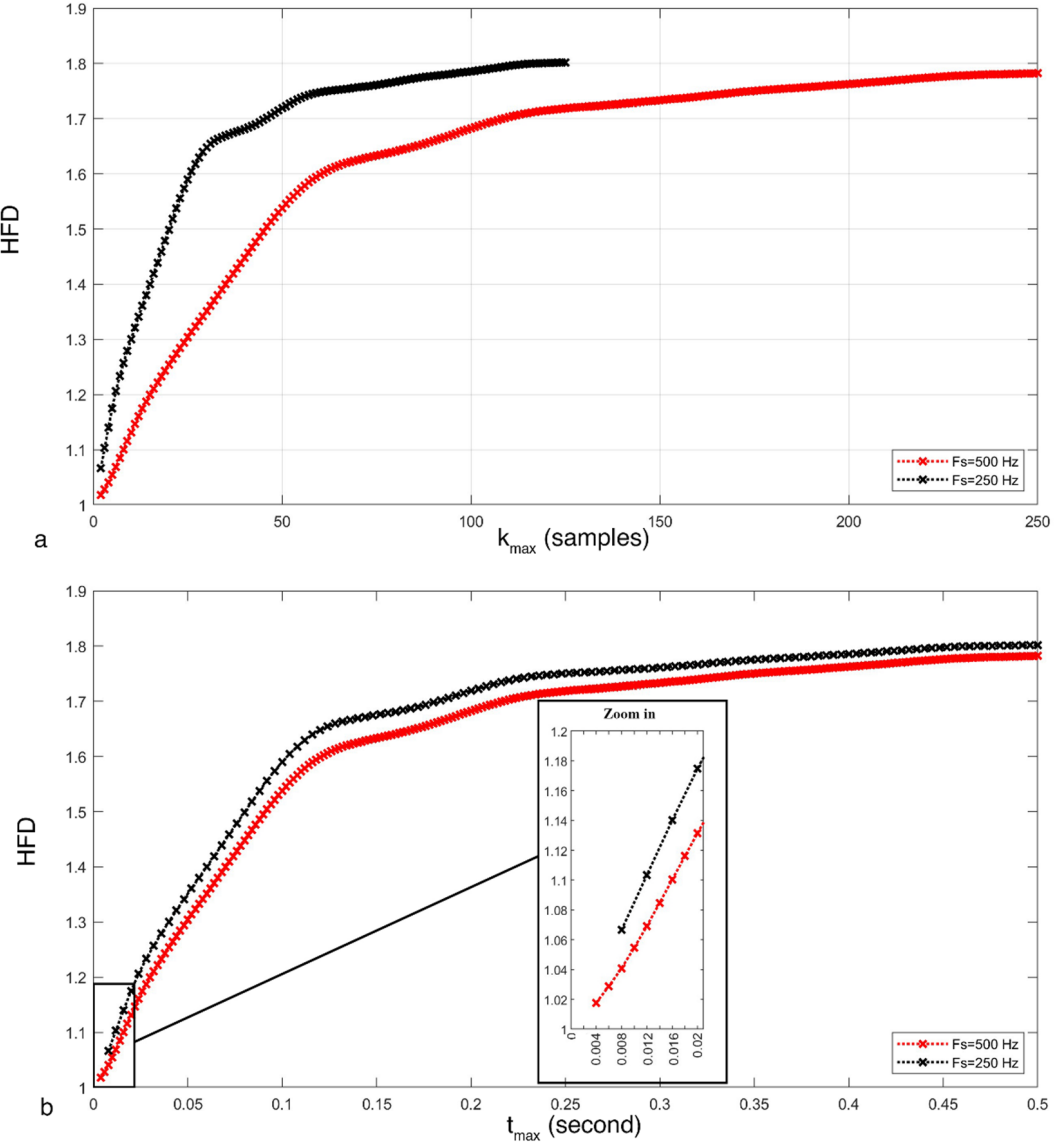
Comparison of HFD values at different sampling frequencies. (**a**) HFD values are plotted against *k*_*max*_ values for sampling frequency (*Fs*) of 500 Hz and 250 Hz, where the lower limit of *k*_*max*_ was set to 2, and the upper limit of *k*_*max*_ was set to cover a maximum time interval of 0.5 s (i.e., 125 for *Fs* = 250 Hz and 250 for *Fs* = 500 Hz). (**b**) HFD values are plotted against maximum time interval (*t*_*max*_) in seconds, with time steps of 0.004 s for *Fs* = 250 Hz, and 0.002 s for *Fs* = 500 Hz.

Figure 4a presents the HFD values against different *k*_*max*_ values for the 500-Hz signal (in red) and 250-Hz signal (in black). As can be seen, the HFD results are relatively similar but do not match on the *k*_*max*_ scale, and are not directly comparable. For example, at *k*_*max*_ = 50, with *Fs* = 500 Hz and 250 Hz, the first evaluates up to a maximum time interval of 0.1 s, while the second evaluates up to 0.2 s. However, this sampling-frequency-specific deviation can be solved by using maximum time intervals rather than samples.

Given that the time step between consecutive samples (*k = 1*) is the inverse of the sampling frequency *1/Fs*, we can describe the scaling in terms of time rather than samples. Specifically, comparisons at scale *k* correspond to a time interval of* K/Fs* seconds, so the HFD analysis spans a finite temporal window from the minimum time interval* 1/Fs* (i.e.,* k* = 1, consecutive samples) to the maximum time interval t_max_=k_max_/*Fs*. Therefore, HFD reflects the scaling behavior of the time series restricted to the temporal window $\:\left[\frac{1}{Fs},{t}_{max}\right]$.

Therefore, we propose presenting HFD using the maximum time interval *t*_*max*_, which removes the main effect of sampling frequency. This is illustrated in Fig. 4b, where HFD values are represented across different *t*_*max*_, for the 500-Hz signal (in red) and 250-Hz signal (in black). The lowest limit of *t*_*max*_ in which HFD can be calculated, corresponds to *k*_*max*_ *= 2* (0.004 s for *Fs* = 500 Hz; 0.008 s for *Fs* = 250 Hz), and the upper limit was previously predefined to 0.5 s. The HFD values of the down-sampled signal exhibit similar trends and characteristics at 250 and 500 Hz sampling frequencies, making the results more comparable than those obtained using the commonly used practice shown in Fig. 4a.

While looking at the zoomed-in results at short *t*_*max*_ (Fig. 4b), we can see that, as expected, the results at a sampling frequency of 500 Hz (indicated by the red line) have smaller time steps than those at 250 Hz (indicated by the black line) (0.002 s and 0.004 s, respectively). Consequently, for the EEG signal with the higher sampling rate, HFD is evaluated with higher temporal density, and the analysis starts earlier.

Additionally, since the sampling frequency of 500 Hz is twice that of 250 Hz, the minimum *k*_*max*_ value for *Fs* = 250 Hz, i.e., *k*_*max*_ = 2, corresponds to *k*_*max*_ = 4 at 500 Hz, ensuring equivalent maximum time intervals. The higher temporal resolution at 500 Hz sampling frequency allows the analysis of smaller time steps (0.002 s), enabling the evaluation of HFD values at additional *t*_*max*_of 0.004 and 0.006 s.

While Fig. 4b shows the HFD-*t*_*max*_ alignment for the example EEG segment, down-sampled to 250 and 500 Hz, we assessed the consistency of this relationship across all 240 ten-second signal segments. While the exact curve shape and HFD values varied across signal segments, on matched *t*_*max*_, the HFD estimates at 250 Hz and 500 Hz exhibited near-identical trends (Spearman’s correlation coefficient (*ρ*): mean = 0.999; min = 0.969; max = 1.000), with values at 250 Hz slightly higher than those at 500 Hz. Despite strong agreement in the trend, a small shift remained between the estimates (mean RMSE ≈ 0.037), which was smaller than the inter-participant variability of HFD in our dataset (SD ≈ 0.061 at 250 Hz and SD ≈ 0.057 at 500 Hz).

## Discussion

The results of this study demonstrate that, when HFD is expressed in terms of *t*_*max*_, the same methodological relationship is observed across all analyzed signal segments. Although the HFD values and the corresponding curve shapes vary across signal segments, reflecting differences in underlying signal characteristics, the overall alignment between estimates is largely preserved. This indicates that the relationship remains consistent across different sampling conditions, despite variations in signal characteristics. It is worth noting that *t*_*max*_ is directly derived from the definition of Higuchi’s algorithm and is not dependent on the specific characteristics of a dataset. It simply represents the conversion of the scale parameter from samples to time (*t*_*max*_ *= k*_*max*_*/Fs*) and is therefore a definitional relationship rather than an empirical quantity. Although this relationship has been demonstrated across different signals and participants in the present study, these analyses are not intended to validate *t*_*max*_ empirically, but rather to illustrate its practical implications in real data.

These findings address a key limitation in the existing literature. While fractal-dimension algorithms have been compared previously and the HFD algorithm has shown promising utility^28^, its interpretation is often limited by inconsistent parameter reporting. In particular, *k*_*max*_ is given only in samples, leaving the underlying time scale analyzed unclear and making comparisons across studies challenging. This becomes even more problematic as HFD is increasingly used in multi-feature and data-fusion frameworks alongside other EEG biomarkers to improve clinical utility. For example, Moaveninejad et al. highlight the increasing use of HFD alongside other EEG biomarkers across neurological and psychiatric applications, arguing that better understanding often requires integrating multiple feature types rather than relying on any single metric^29^. However, interpreting multiple features is considerably more challenging, making the *t*_*max*_ approach even more necessary.

Accordingly, we show that by presenting the maximum time interval (*t*_*max*_), HFD values can be compared across studies. For example, the studies by Põld et al.^15^ (*k*_*max*_ = 40, *Fs* = 400 Hz), and Wu et al.^30^ (*k*_*max*_ = 50, *Fs* = 500 Hz), both analyzing the maximum time interval of 0.1 s, are comparable. Similarly, a comparison can be performed between the study by Croce et al.^26^ (*k*_*max*_ = 8, *Fs* = 125 Hz), and Zappasodi et al.^31^ (*k*_*max*_ = 16, *Fs* = 256 Hz), both analyzing the maximum time interval of 0.06 s. However, comparing studies conducted at different maximum time intervals is rather arbitrary. These findings underscore that the implications of *t*_*max*_ *= k*_*max*_*/Fs* selection merit explicit consideration and should be reflected in future methodological guidelines.

Although the HFD values plotted against the maximum time interval in Fig. 4b are more aligned, a small shift in HFD values persists when comparing results from the same signal at different sampling rates. This is because *t*_*max*_ approach does not compensate for differences in the temporal resolution. Specifically, HFD evaluates the scaling relationship starting from the minimum scale (1/*Fs*, which also defines the temporal resolution for calculations), up to the scale defined by *t*_*max*_. That minimum scale and the overall temporal resolution always influence the outcome of the HFD method. Consequently, some residual differences at different sampling rates are expected. However, as shown in this study, the magnitude of error (RMSE) is smaller than the inter-participant variability (SD), indicating that the *t*_*max*_ approach preserves between-participant differences and improves comparability across studies.

While interpreting the HFD results, the maximum time interval (*t*_*max*_) determines the frequency range effectively represented in the scaling analysis. Consider an oscillatory component with frequency *f* and period (*T = 1/f*). For this component to be represented in the analysis, the set of evaluated scales must span a sufficient portion of its characteristic timescale. In particular, the maximum time interval must be at least as long as half its period *(i.e.*,* t*_*max*_ *≥ T/2 ≥ 1/(2f))* to adequately capture its variation. If this condition is not met, the oscillation is insufficiently represented, and its contribution to the estimated scaling behavior is reduced. Rearranging this condition yields (*f ≥ 1/(2t*_*max*_*))*, indicating that only frequencies above this threshold are effectively represented. Consequently, *t*_*max*_ defines the minimum frequency that HFD is focused on (*f*_*min*_ *= 1/(2t*_*max*_)), while the highest frequency component is determined by the sampling frequency. This should be viewed as an interpretative boundary rather than a strict spectral cutoff imposed by the HFD algorithm.

As an example, in the study by Croce et al.^26^, with a sampling frequency of 125 Hz and *k*_*max*_ = 8, the maximum time interval is *t*_*max*_ = 0.064 s, which represents half the period of the slowest oscillation in the signal. Consequently, the minimum frequency focused by HFD algorithm corresponds to 7.81 Hz. In other words, calculating HFD at *t*_*max*_ = 0.064 s can characterize all the frequencies in the signal from 7.81 Hz and above. This means frequencies below 7.81 Hz are not in focus while evaluating the fractal dimension. Consequently, the HFD analysis focuses on the alpha and beta bands, while contributions from the delta band and most of the theta range are not specifically targeted by the analysis.

Table 1 presents multiple studies as examples, illustrating how different parameter choices change the frequency range that HFD focuses on. While some studies focus on high-frequency bands, others extend the analysis to lower frequencies, thereby covering a broader spectrum. This variability suggests that HFD is not uniformly focused on the full EEG spectrum; instead, it reflects the specific temporal and spectral window defined by *t*_*max*_. Consequently, HFD values differ across studies not only because of physiological differences, but also because of methodological factors, including the frequency band focused on by HFD and the temporal resolution. As a result, studies that rely on different spectral ranges may report divergent results, even when there is no underlying physiological difference or actual change in the subject’s condition. As such, cross-study comparisons require careful attention to these methodological settings to ensure valid interpretation.

**Table 1:** Example studies showing variability in the frequency band(s) focused on by HFD.

Study	Fs (Hz)	k_max_	t_max_ (s)	f_min_ (Hz)	Focused frequency band(s)	Reported HFD in healthy participants (mean ± SD)
Čukić et al. (2020)^14^	1000	8	0.008	62.5	High gamma+	1.0196 ± 0.0002
Jacob et al. (2019)^32^	500	6	0.012	41.67	Low gamma+	1.60 ± 0.09
Päeske et al. (2023)^27^	200	8	0.040	12.5	Beta +	–
Zappasodi et al. (2015)^31^	256	16	0.0625	8	Alpha+	1.484 ± 0.102
Põld et al. (2021)^33^	400	50	0.125	4	Theta+	1.697 ± 0.049 1.700 ± 0.053 (two sessions)
Parbat et al. (2021)^34^	256	50	0.195	2.56	Delta+	–

While HFD has been shown to correlate with conventional band-power measures^27^, it provides complementary information beyond these linear measures. As a time-domain scaling metric, HFD is sensitive to the signal’s temporal structure, including irregularity and nonstationarity, which spectral measures cannot reveal. HFD has shown better discrimination between depressive and healthy groups than spectral power features alone^9,11,30^.

Zappasodi et al. demonstrated that HFD variations are bound to changes in the alpha and beta power^31^. This is consistent with our finding that the choice of *t*_*max*_ sets the temporal scales for the estimation and modulates the spectral region to which HFD is focused. Since *t*_*max*_ determines the temporal scale analyzed, varying this parameter does not just change the numerical HFD value; it may inadvertently shift the focus to different functional networks^35^. A higher HFD in one study might simply reflect a sampling setup that emphasizes high-frequency local processing over low-frequency network integration.

In addition, a limitation in the present study should be considered. As a non-linear method, HFD is sensitive to different pre-processing steps. In the current study, the signal segments were pre-processed by a zero-phase lowpass filter with a cutoff frequency of 47 Hz. Although some argue that filtering should be avoided to preserve the fractal characteristic of the signal, the first signal modification is already performed by the device’s analog filter. One must consider that non-linear methods are sensitive to preprocessing steps.

## Conclusion

This study demonstrates that HFD results depend on both sampling frequency and the parameter *k*_*max*_, which limits direct comparison across studies. By expressing HFD values in terms of the maximum time interval (*t*_*max*_ *= k*_*max*_*/Fs)* rather than *k*_*max*_, we provide a consistent time-scale representation that addresses this limitation. This representation enables direct comparison across studies that analyze the same time intervals and improves the consistency of HFD values.

Additionally, we demonstrated that the maximum time interval (*t*_*max*_) determines the frequency range that HFD focuses on, thereby providing a basis for results interpretation and parameter selection. This is important as previous studies have differed in their spectral focus, with some examining high-frequency bands and others using a broader spectrum that includes both low- and high-frequency bands.

Overall, these findings underscore the importance of explicitly expressing HFD in terms of the maximum time interval, which provides a foundation for comparing studies. Future research should explore whether targeting specific frequency bands can further enhance HFD’s potential as a biomarker for neurological and mental conditions.

## Data Availability

Data are available upon request from the corresponding author.
